# Wound Healing Properties and Antimicrobial Effects of *Parkia clappertoniana* Keay Fruit Husk Extract in a Rat Excisional Wound Model

**DOI:** 10.1155/2022/9709365

**Published:** 2022-07-23

**Authors:** Dominic Nkwantabisa Kuma, Alex Boye, Godwin Kwakye-Nuako, Yaw Duah Boakye, Justice Kwaku Addo, Ernest Amponsah Asiamah, Eugene Agyei Aboagye, Orleans Martey, Mainprice Akuoko Essuman, Victor Yao Atsu Barku

**Affiliations:** ^1^Department of Biomedical Sciences, School of Allied Health Sciences, College of Health and Allied Sciences, University of Cape Coast, Cape Coast, Ghana; ^2^Department of Medical Laboratory Science, School of Allied Health Sciences, College of Health and Allied Sciences, University of Cape Coast, Cape Coast, Ghana; ^3^Department of Pharmaceutics, Faculty of Pharmacy and Pharmaceutical Sciences, Kwame Nkrumah University of Science and Technology (KNUST), Kumasi, Ghana; ^4^Department of Chemistry, School of Physical Sciences, College of Agriculture and Natural Sciences, University of Cape Coast, Cape Coast, Ghana; ^5^Department of Forensic Science, School of Biological Sciences, University of Cape Coast, Cape Coast, Ghana; ^6^Specialist Pathologist, Manhyia District Hospital, Kumasi, Ghana; ^7^Department of Pharmacology, Center for Plant Medicine Research (CPMR), Mampong-Akuapem, Eastern Region, Ghana

## Abstract

**Background:**

*Parkia clappertoniana* Keay (Family: Fabaceae) (*P. clappertoniana*) fruit husk is commonly used in northern Ghana for wound treatment. However, this folk claim remains to be confirmed scientifically.

**Objective:**

This study investigated wound healing and antimicrobial effects of *P. clappertoniana* fruit husk extract (PCFHE) by using excision wound model in rats.

**Materials and Methods:**

After preparation and phytochemical analysis of PCFHE, it was reconstituted in purified water and emulsifying ointment yielding a wound healing formula (0.3, 1, and 3%). Excision wounds were established in healthy male Sprague-Dawley rats (aged 8-10 weeks; weighing 150–200 g). Rats were randomly assigned into six groups (model, 1% silver sulfadiazine [SSD], vehicle, and PCFHE [0.3, 1, and 3%, respectively]) and topically treated daily until complete wound healing. The endpoints (period of epithelialization, wound contraction, collagen content, erythema index, oedema index, inflammatory cell infiltration, and antimicrobial activity) were assessed for all groups. Minimum fungicidal concentration (MFC), minimum inhibitory concentration (MIC), minimum bactericidal concentration (MBC), and time-kill were assessed.

**Results:**

Quercetin and catechin were detected in PCFHE. Compared to model and vehicle groups, PCFHE-treatment groups improved wound healing and antimicrobial (MBC, MFC, and MIC) endpoints. PCFHE demonstrated bacteriostatic and fungicidal effects against identified wound contaminants (*Pseudomonas aeruginosa*, *Klebsiella pneumoniae*, *Escherichia coli*, and *Candida albicans*).

**Conclusion:**

*P. clappertoniana* fruit husk possesses wound healing and antimicrobial effects in excisional wounds in rats that confirms its folk use, and the reported pharmacological properties of PCFHE are attributable to its quercetin and catechin phyto-constituents.

## 1. Introduction

Wounds result from physical aberration of epithelial tissues such as the skin. Such epithelial aberrations commonly do not only disrupt barrier function but also may lead to bleeding, coagulation of blood vessels, complement activation, and inflammatory reaction [1]. Wounds may complicate human health and well-being including disfiguration of external body, impaired body function, decrease in confidence level of people with life-long body deformities and wound-related morbidities [2]. Also, chronic nonhealing wounds afflict patients with varied degrees of pain, discomfort, and distress while at the same time putting pressure on care givers and the healthcare system. For instance, it is estimated that annually chronic nonhealing wounds resulting from diabetes mellitus cost a total of US$25 billion, yet the number of affected patients keeps increasing (6.5 million) in tandem with potential risk factors (diabetes mellitus and chronic diseases) [3]. Globally, wound treatment/management is estimated to attract a market value of US$320 billion, and this conservative estimate is projected to increase at a rate of 7% per annum [2]. Example, diabetic ulcer requires over US$49,000 as cost of treatment [4]. In the UK, the National Health Service indicates that annually between £2.5 and 3.1 billion is spent on wound treatment, and this amount accounts for almost 3-4% of the healthcare budget [2]. Increased hospital attendance and hospitalizations have in part been attributed to chronic nonhealing wounds aggravated by resistant wound contaminants. One major factor implicated in the development of chronic nonhealing wounds is microbial contamination. Organisms such as *Pseudomonas aeruginosa*, *Staphylococcus aureus*, *Acinetobacter* spp., vancomycin-resistant *Staphylococcus aureus* (VRSA), and methicillin-resistant *Staphylococcus aureus* (MRSA) are common contaminants of wounds [5]. Wound-related burden globally and specifically in the UK may not be significantly different from what pertains in Africa, particularly given the poor healthcare systems and resource constraints in most African countries.

Conventionally available wound healing therapies include irrigation, debridement, use of antibiotics, proteolytic enzymes, and tissue grafts [6]. However, the available conventional wound healing therapies are fraught with setbacks ranging from invasiveness, high-cost of treatment, and sometimes. treatment failure [7, 8]. It is widely acknowledged that setbacks associated with conventional wound healing therapies, particularly, treatment failure and high cost of treatment, have necessitated a switch from reliance on conventional wound healing therapies to a much cheaper and readily available traditional wound healing approaches dominated by plants and plant-derived products. For instance, wound healing potentials of a number of plants including *Pistacia atlantica*, *Zataria multiflora*, *Trifolium repens*, *Quercus infectoria*, and *Salvia officinalis* have been confirmed in preclinical studies through use of *in vitro* and *in vivo* wound models [9–13]. Most often, plant-based wound healing therapies are inspired by the belief systems and ethnobotanical heritage of the people who use such wound healing therapies. Interestingly, some plant species in the genus Parkia find use in folklore of many cultures in view of its suspected wound healing properties. The genus Parkia (family: Fabaceae; subfamily: mimosoideae) embodies about 34 species, which are mostly perennial plants. Ecologically, these plant species are distributed in the tropics (e.g., Africa and some parts of Asia) and neotropical (South America, Central America, Caribbean Islands, and South north America) regions of the world [14, 15]. Some of the common species in the genus Parkia includes *Parkia clappertoniana*, *Parkia biglobosa*, *Parkia speciosa*, *Parkia bicolor*, *Parkia javanica*, *Parkia filicoideae*, *Parkia biglandulosa*, *Parkia pendula*, and *Parkia platycephala* [14]. Aside their use as important food source, they are also used in folklore. For example, plant species in the genus Parkia are reported to be used traditionally in the treatment of wounds, diarrhoea, diabetes mellitus, cough, hypertension, chronic piles, measles, and conjunctivitis [14]. A number of secondary plant metabolites including terpenoids, phenolic acids, volatile compounds, and flavonoids (aglycone and glycosides) have been identified in the genus Parkia, and all the reported pharmacological properties of plant species in the genus Parkia have been attributed to these secondary plant metabolites [14, 16].

Until recently, northern Ghana was known for its guinea worm endemicity. Local people treat guinea worm infections by physical removal of the adult worm from the infected skin area, a practice which leaves behind sores of various kinds. To treat such sores and cutaneous wounds, local people use the fruit husk of *P. clappertoniana* in crude forms as a potent wound healing agent. In view of the ethnobotanical relevance of *P. clappertoniana*, a number of studies have assessed some of its folk claims such as gastro-protection, reno-protection, antimicrobial, antiplasmodial, antimolluscicidal, antihypertensive, antidiabetic, and antisickling effects [15, 17–20]. Although, use of *P. clappertoniana* fruit husk in northern Ghana as a crude wound healing therapy is quite common and uneventful, it remains to be scientifically verified and validated. The present study assessed wound healing and antimicrobial effects of *P. clappertoniana* fruit husk extract (PCFHE) as a first attempt to scientifically verify its uneventful use as a wound healing agent. By using excision wound model in rats and sensitivity testing of wound contaminants to PCFHE, it demonstrated wound healing efficacy and bacteriostatic effects against common resistant wound contaminants such as *Pseudomonas aeruginosa*, *Klebsiella pneumoniae*, *E. coli*, and *Candida albicans*.

## 2. Materials and Methods

### 2.1. Chemicals and Reagents

Chemicals and reagents used in the study included but not limited to chloroform, ethanol, acetic anhydride, methanol, dichloromethane (Merck BDH, Poole, UK), ferric chloride, sulphuric acid, sodium hydroxide, trifluoroacetic acid, formic acid, ammonia, ammoniacal alcohol solution, and Dragendorff's reagent, (Sigma-Aldrich, London, UK). All other chemicals and reagents used in this study met analytical grade.

### 2.2. Collection, Identification, and Authentication of Plant Parts

The fruit husk of *Parkia clappertoniana* was collected from Chinderi, Krachi East District in the Oti Region of Ghana. The fruit husk was identified and authenticated by a plant taxonomist at the herbarium unit, School of Biological Sciences, University of Cape Coast, Cape Coast, Ghana, where a voucher specimen (UCC/SBS/P133) was deposited.

### 2.3. Preparation of Ethanol Extract of *Parkia clappertoniana* Fruit Husk (PCFH)

The fruit husk of *P. clappertoniana* were washed and air-dried until constant dry weight was attained. The dried fruit husk was milled into fine powder by using a milling machine (Vivekananda Madras, U.S.A) and a blender (Chefman, England, 1985). The powdered fruit husk was further sieved to remove particulates from the mixture resulting in a fine fruit husk powder. The fine fruit husk powder (weighing 342.25 g) was extracted with 70% ethanol using cold maceration, filtered, and then, evaporated on water bath to obtain PCFH.

### 2.4. High-Performance Liquid Chromatography (HPLC) Analysis on PCFH

HPLC analysis on PCFH was conducted according to a previously described method [21] with modification. Briefly, a 10 mg of PCFHE was weighed into a beaker containing water (5 mL). It was transferred into a 10 mL volumetric flask and topped up with deionized water to the 10 mL mark. The mixture was sonicated for 10 minutes. The solution was filtered into a 2 mL vial with a 0.45 *μ*m membrane filter before being loaded into the HPLC system for analysis. A 10 mg each of quercetin and catechin (standards) were weighed separately into two beakers and 5 mL distilled water added to each. Each was transferred into a 10 mL volumetric flask and topped up with deionized water to the 10 mL mark, then sonicated for 10 minutes in each case. The solutions were filtered into a 2 mL vial with a 0.45 *μ*m membrane filter before being loaded into the HPLC system for analysis. The system used was a Shimadzu LC-20 AD HPLC system, equipped with a model LC-20 AV pump, UV detector SPD-20AV, Rheodyne fitted with a 5 mL loop, lab solution, and autoinjector SIL-20 AC. Agilent's Zorbax 300B-C18 (4.6∗250 mm, 5 *μ*m) column was used. At room temperature, the elution was carried out using gradient solvent systems at a flow rate of 1 mL/min. 0.05% trifluoroacetic acid (A) and methanol (B) made up the mobile phase. It lasted for 60 min. The sample injection volume was 5 mL, and the UVevis detector wavelength was set to 275 nm. The HPLC chromatogram developed for the extracts was compared to those of the standards developed under the same conditions.

#### 2.4.1. Confirmatory Test for Catechin in PCFHE

A white match stick was moistened with an aqueous solution of PCFHE (2 mg/mL). The moistened match stick was then allowed to dry completely and then dipped in conc. HCl (37% *w*/*v*) after which it was gently warmed. Appearance of pink coloration confirmed the presence of catechins.

#### 2.4.2. Confirmatory Test for Quercetin in PCFHE

To an aqueous solution of PCFHE (2 mg/mL), few fragments of magnesium turning was added and drops of conc. HCl (37% *w*/*v*) was added. A crimson red appearance after few minutes confirmed the presence of quercetin.

### 2.5. Preparation of PCFHE-Reconstituted Wound Healing Formula (WHF)

PCFHE, purified water, and emulsifying ointment were weighed according to a predetermined proportion (Table 1) by using an electronic balance. A mixture of PCFHE and purified water and emulsifying ointment were separately heated on a heating slab at 70°C. Temperatures were closely monitored using a thermometer. By using a homogenizer, the heated PCFH/purified water mixture and the emulsifying ointment were mixed (Table 1) and swirled until a uniform mixture was formed. Subsequently, the uniform mixtures were swirled intermittently until there was formation of a cream which was code-named PCFHE-reconstituted wound healing formula (PCFHE-RWHF). PCFHE-RWHF formulas were transferred into labeled containers according to their strengths and stored in a refrigerator at 4°C until use.

### 2.6. Acquisition and Care of Animals

Healthy 8-10 week old male Sprague-Dawley rats weighing (150–200 g) were purchased from the Noguchi Memorial Institute for Medical Research (NMIMR), University of Ghana, Legon, and transported to the animal house of the Department of Pharmacology, Faculty of Pharmacy and Pharmaceutical Sciences, Kwame Nkrumah University of Science and Technology (KNUST), where the study was conducted. The rats were fed on pelleted feed (AGRICARE Ltd, Kumasi, Ghana) and allowed free access to clean water. Rats were kept under ambient conditions of light/dark, humidity, and room temperature throughout the study. The “Principles of laboratory animal care” (NIH publication No. 85-23, revised 1985) were followed, as well as specific national laws where applicable and also all activities performed during the studies conformed to accepted principles for laboratory animal use and care (EU directive of 1986: 86/609/EEC).

### 2.7. Excision Wounding

Excisional wounds were created in rats per a previously described method [9, 22] with some modification. Briefly, rats were anaesthetized using chloroform (100 ppm; *v*/*v*) in inhalation chambers prior to creation of wounds. Furs on the dorsum of rats were shaved with an electric clipper, and the area of the wound to be created was outlined on the back of the rats with methylene blue using a circular stainless steel stencil. A full thickness of excision wound of 2 cm in width (circular area of 4 cm^2^) was created along the markings using toothed forceps, a surgical blade, and a pointed scissor. All the surgical interventions were carried out under sterile conditions. After 24 h of wound creation, ~0.1 g of each agent, i.e., PCFHE-wound healing formula, 1% silver sulfadiazine (SSD), and vehicle (emulsifying ointment) were topically applied to gently cover the wounded area once daily until complete wound healing. Wound area and wound contraction were monitored every 48 hours until complete wound healing was achieved for all groups.

### 2.8. Animal Grouping and Treatment

A total of 30 excision wounded rats were randomly reassigned into six groups of five rats each as follows: model group (excision wounds + no treatment), control (excision wounds +1% SSD), vehicle group (excision wounds + emulsifying ointment), and PCFHE (excision wounds +0.3, 1, and 3%, respectively). All the wounds received daily standard wound cleansing with 0.9% normal saline prior to the topical application of treatments (0.1 g/unit wounded area [4cm^2^]/day). Any rat showing a wound hematoma or wound infection was immediately euthanized with an overdose of ketamine hydrochloride (200 mg/kg).

### 2.9. Measurement of Wound Area

The diameters of the excised wounds were measured immediately after wound creation and subsequently every 48 hours postwounding until wounds in all groups were completely healed. Also, period of epithelialization was monitored for each group. Additionally, wound areas of rats were photographed every 72 hours for the first 15 days for all groups. Wounds were measured by gently covering the wound area with a white tissue paper which traces the outline of the wound area. The length and breadth of the traced wounded areas were then measured using calipers. The area of wound contraction was then calculated according to a previously described method [11] as shown below:

(1) $$\begin{array}{c} \%\text{wound contraction}=\frac{\text{Day }0\text{ wound area}-\text{Day }Z\text{ wound}}{\text{Day }0\text{ wound area}}\times 100, \end{array}$$

where *Z* is the day of wound area measurement other than day 0.

### 2.10. Swabbing of Wounds

Before cleansing of wounds with 0.9% normal saline, swab sticks were used to swab wounds and the tips of swab sticks immediately dipped in peptone water. Swabs dipped in peptone water were transported to the laboratory under refrigeration at 2-8°C. All swab samples were examined on the day of collection. The swabs were vortex mixed to ensure release of contaminating microorganisms into the diluent for culture, microbial identification and antimicrobial sensitivity test.

### 2.11. Isolation of Wound Tissue

Upon complete wound healing, rats were sacrificed by intramuscular injection of ketamine hydrochloride (200 mg/kg). The wound tissues were excised 24 h after complete wound healing and transferred into 10% neutral buffered formalin (100 mL 37% formaldehyde, 6.5 g sodium phosphate, dibasic (Na_2_HPO_4_), 4 g sodium phosphate, monobasic (NaH_2_PO_4_) in 1 L distilled water) for histological preparation and assessment.

### 2.12. Histological Assessment of Healed Wound Tissues

Healed wound tissue sections were made according to a previously described method [23] with some modifications. Briefly, healed tissue samples were fixed in 10% neutral buffered formalin, successively dehydrated using graded alcohol, cleared in xylene, and subsequently embedded in paraffin. A semiserial 4-micrometer (𝜇m) sections were made using microtome (HM-355S Automatic Microtomes Thermo Scientific); stained with Harris hematoxylin and eosin (H&E) and permanently mounted on microscopic slides using DPX; cover slipped and then observed under a light microscope (Zeiss, Germany). Field were viewed and images captured using an optical microscope (Zeiss, Germany) coupled to a high resolution camera (AmScope, California) and analyzed using AmScope Software 2020.

### 2.13. Collagen Staining

After preparation of healed wound sections and waxing, each section was dewaxed and hydrated. Picro-sirius red (0.1 g of sirius red +100 mL of saturated aqueous picric acid) and acidified water (5 mL glacial acetic acid +1 L distilled water) were mixed and used to stain the sections for 10 minutes. Sections were washed in two changes of acidified water, transferred, and stained in Van Gieson solution (saturated aqueous picric acid +1% acid fuchsine) for 1 h. Sections were dehydrated in three changes of 100% ethanol. Finally, sections were cleared in xylene and mounted in resinous medium for microscopic examination.

### 2.14. Quantification of Collagen

**Figure 1:**
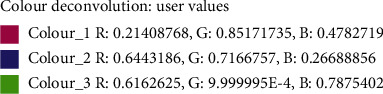
Threshold values of color deconvolution for quantification of collagen content.

Micrographs of healed wound tissues were processed for collagen content estimation using ImageJ analysis. By using the color deconvolution plug-in in ImageJ 1.53c and an optimized “user values” vector, the components of the histological stains (picro-sirus red and Van Gieson) were separated. The processed images were inverted and subjected to automatic thresholding. The percentage threshold values (Figure 1) for the red and blue/violet stains were then recorded.

### 2.15. Skin Irritation Test

Modified occluded dermal irritation test as previously described [24] was used to assess dermal toxicity of PCFHE. There are a total of six rats, three rats each for PCFHE and negative control (5% NaOH). After chloroform anesthesia, a defined area of the dorsal region (1 cm from the midline of the vertebral column) of each rat was shaved using a clipper and marked out. Upon complete recovery of animals from anesthesia, test agents were topically applied to shaved areas (6 cm^2^) of each rat and covered with a dressing gauze held firmly in place by a nonirritating adhesive tape and tied across the diameter of the back of each rat with an elastic bandage. After 24 h of exposure period, the elastic bandage, the adhesive plaster, the plastic sheet, and the gauze were carefully removed, and the test site was washed with distilled water. Each rat was examined for the presence of erythema and oedema according to Draize dermal irritation scoring system at time intervals (0, 6, 24, 48, and 72 h). The degree of erythema and oedema was determined based on the scoring guide shown below (Table 2).

### 2.16. Test Organisms

From the swab cultures, identified contaminating organisms informed the selection of test organisms for sensitivity studies on PCFHE. Gram positive, Gram negative, and a fungus were selected for the susceptibility testing, minimum inhibitory concentration (MIC), and minimum bactericidal/fungicidal concentration (MBC/MFC) determination as well as the time-kill kinetics studies. Three (3) typed strains of bacteria (*Escherichia coli* ATCC 25922, *Staphylococcus aureus* ATCC 25923, *Bacillus subtilis NTCC10073*, and two clinical strains of bacteria (*Pseudomonas aeruginosa* and *Klebsiella pneumonia*) and a clinical fungus (*Candida albicans*) were obtained from the Department of Biological Sciences, Kwame Nkrumah University of Science and Technology (KNUST), Kumasi, Ghana. They were maintained on 20 mL nutrient agar slants containing 30% glycerol and stored at -4°C in a frost-free freezer in the Microbiology Laboratory of Department of Pharmaceutics, Kwame Nkrumah University of Science and Technology (KNUST), Kumasi, Ghana [26]. These strains were subcultured aseptically into a freshly prepared 10 mL nutrient broth and incubated for 24 h prior to their use at 37°C and 25°C for test bacteria and fungus, respectively. The identity of each test organism was confirmed before use by culturing on the specific selective media followed by biochemical characterization.

### 2.17. Standardization of Test Organisms

The number of viable cells in a given suspension of test organisms was determined according to a previous method [27]. Dilutions of 1 in 10, 1 in 10^2^, 1 in 10^3^, and 1 in 10^4^ of 24 h broth culture of the organisms were prepared and the absorbance determined at 420 nm. The colony forming units (CFU) in the dilutions made was determined by direct plate count method using plate count agar after incubation at 37°C for 24 h. A graph of the log of CFU/mL was then plotted against the absorbance to obtain a calibration curve. The calibration curve was used to determine the number of viable cells for a given suspension of test organisms after the determination of its absorbance. Inocula with high number of cells were diluted with normal saline (0.9% *w*/*w*) to obtain the absorbance that gives the required number of viable cells.

### 2.18. Susceptibility Testing (Agar Well Diffusion Method)

The sensitivity of test organisms to PCFHE was assessed by using agar diffusion method [28] with some modifications. Briefly, nutrient agar and potato dextrose agar were used for the determination of the antibacterial and antifungal activities, respectively. PCFHE (1 g) was weighed and dissolved in 5 mL of distilled water to produce a stock solution of 200 mg/mL. Twofold serial dilutions were performed from the stock to produce concentrations of 12.5, 25, 50, and 100 mg/mL. Twenty milliliters each of nutrient agar and potato dextrose agar were seeded with 100 *μ*L (1 × 10^6^ CFU/mL) of test bacteria and fungus, respectively, and transferred aseptically into sterile petri dishes. In each of these plates, six equidistant wells (10 mm) were cut out using sterile cork borer (No. 5), and wells were filled with 100 *μ*L of PCFHE (12.5, 25, 50, and 100 mg/mL, respectively) and allowed to diffuse into seeded agar plates for 1 h at 25°C. The zones of growth inhibition (including diameter of well) were measured after 24 h of incubation at 37°C for bacteria and 24 h postincubation at 28°C for fungus. The experiment was performed in triplicate, and the mean zones of growth inhibition were determined. Ciprofloxacin and fluconazole were used as reference drugs against test bacteria and fungus, respectively. The same procedure was repeated for PCFHE at concentrations of 1.25, 2.5, 5, and 10 mg/mL. PCFHE (100 mg) was weighed and dissolved in 5 mL of distilled water to produce a stock solution of 20 mg/mL.

### 2.19. Determination of Minimum Inhibitory Concentration (MIC)

The minimum inhibitory concentrations of PCFHE was determined by the broth dilution method as described previously [28] with some modifications. Briefly, a 96 well microtitre plates were each filled with 100 *μ*L of double strength nutrient broth. PCFHE (200 mg) was weighed and dissolved in 5 mL of distilled water to produce a stock solution of 40 mg/mL. A specified volume (10 to 50 *μ*L) of the stock was added to each well to obtain a serial two-fold dilution of PCFHE in each well with concentrations within the range 0.1 to 10 mg/mL. An inoculum size of 20 *μ*L (1.0 × 10^6^ CFU/mL) of test organisms were added to the appropriately labeled wells, and activity was determined against test organisms after incubating at 37°C. After 24 h postincubation, the minimum inhibitory concentration (MIC) was determined as the lowest concentration of extract that inhibited growth which was indicated by the absence of purple coloration upon the addition of 20 *μ*L of 3-(4, 5-dimethylthiazol-2-yl)-2, 5-diphenyltetrazolium bromide (MTT) to the microtiter wells and incubated at 37°C for 30 min. Ciprofloxacin (0.01 to 1.0 *μ*g/mL) and fluconazole (1.0 to 10.0 *μ*g/mL) were used as reference antimicrobial agents for bacteria and fungi, respectively.

### 2.20. Determination of Minimum Bactericidal and Fungicidal Concentrations

The minimum bactericidal and fungicidal concentrations of PCFHE were determined according to a previous method [29] with some modification. Briefly, a 96 well microtiter plates were each filled with 100 *μ*L of double strength nutrient broth. PCFHE (2 g) was weighed and dissolved in 5 mL of distilled water to produce a stock solution of 400 mg/mL. A specified volume (10 to 50 *μ*L) of the stock was added to each well to obtain a serial twofold dilution of PCFHE in each well with concentrations within the range of 1.0 to 100.0 mg/mL. An inoculum size of 20 *μ*L (1.0 × 10^6^ CFU/mL) of test organisms was added to the appropriately labeled wells, and activity was determined against test organisms after incubating at 37°C. After 24 h postincubation, aliquots (100 *μ*L) were taken from the various wells and inoculated into freshly prepared 1 mL nutrient broth. The inoculated nutrient broths were incubated at 37°C for 24 h. The MBC or MFC was determined as the least concentration of extract at which no purple coloration was observed upon the addition of 20 *μ*L of MTT (1.25 mg/mL) and incubation at 37°C for 30 min. The test was performed in triplicate to validate the results.

### 2.21. Time-Kill Assay

The time-kill kinetic studies on PCFHE were done according to a modified previous method [26, 30]. A 1 × MIC, 2 × MIC, and 4 × MIC of PCFHE were prepared in test tubes containing 10 mL double strength nutrient broth. An inoculum size of 1 mL (1.0 × 10^6^ CFU/mL) was added to each test tube. The tubes were incubated at 37°C and 1 mL of the medium was taken at time intervals of 1, 2, 3, 4, 5, 6, and 24 h and inoculated aseptically into 20 mL nutrient agar which was subsequently transferred into a sterile petri dish. The plates were then incubated at 37°C for 24 h. A control test was performed for each test organism without PCFHE. The CFU of each test organism was determined. The procedure was performed in triplicate, and the average CFU/mL was calculated. A graph of the log CFU/mL was then plotted against time.

### 2.22. Statistical Analysis

GraphPad Prism 7 software (GraphPad Software, San Diego, California, U.S.A.) was used in analyzing data. Data was presented as mean ± standard deviation (SD). Mean differences between treatment groups were analyzed by using one-way analysis of variance (ANOVA) and Dunnett's multiple comparison test. In all analyses, *P* ≤ 0.05 was considered statistically significant.

## 3. Results

### 3.1. Phytochemical Profile of PCFHE

**Figure 2:**
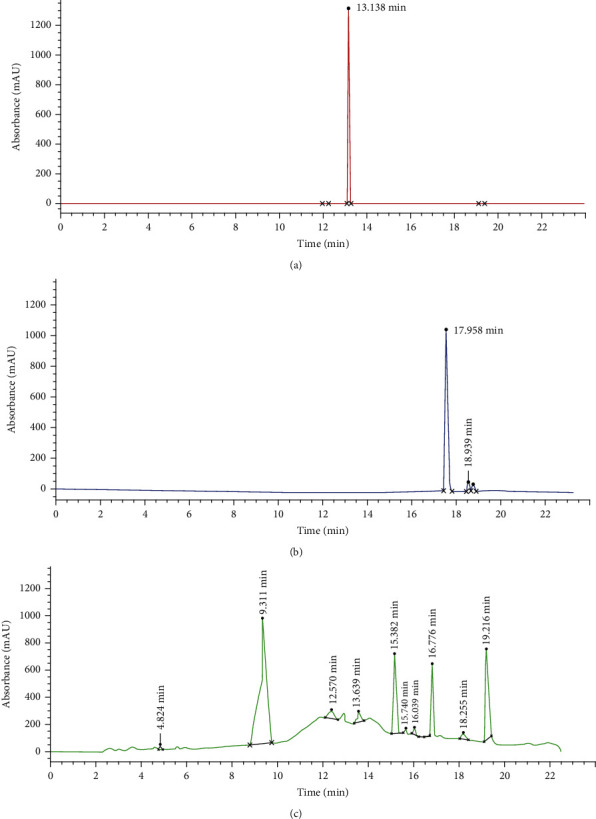
HPLC chromatogram of PCFHE. Catechin (a), quercetin (b), and PCFHE (c). PCFHE: *P. clappertoniana* fruit husk extract.

By using standard phytochemical methods, phenols, flavonoids, tannins, glycosides, saponins, and terpenoids were qualitatively detected in PCFHE (Table 3). Further, HPLC analysis showed that PCFHE contains a number of compounds including catechin and quercetin (Figure 2).

### 3.2. Effect of PCFH Wound Healing Formula on Period of Epithelialization

**Figure 3:**
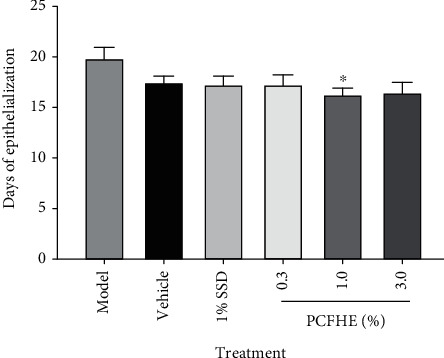
Effect of *P. clappertoniana* fruit husk extract- (PCFHE-) reconstituted wound healing formula on period of epithelialization of excisional wounds in rats. Data are expressed as mean ± SD, *n* = 5. Differences in mean between treatment groups were done by using one-way analysis of variance followed by Tukey's test. ^∗^*P* < 0.05 (model vs. treatment groups).

**Figure 4:**
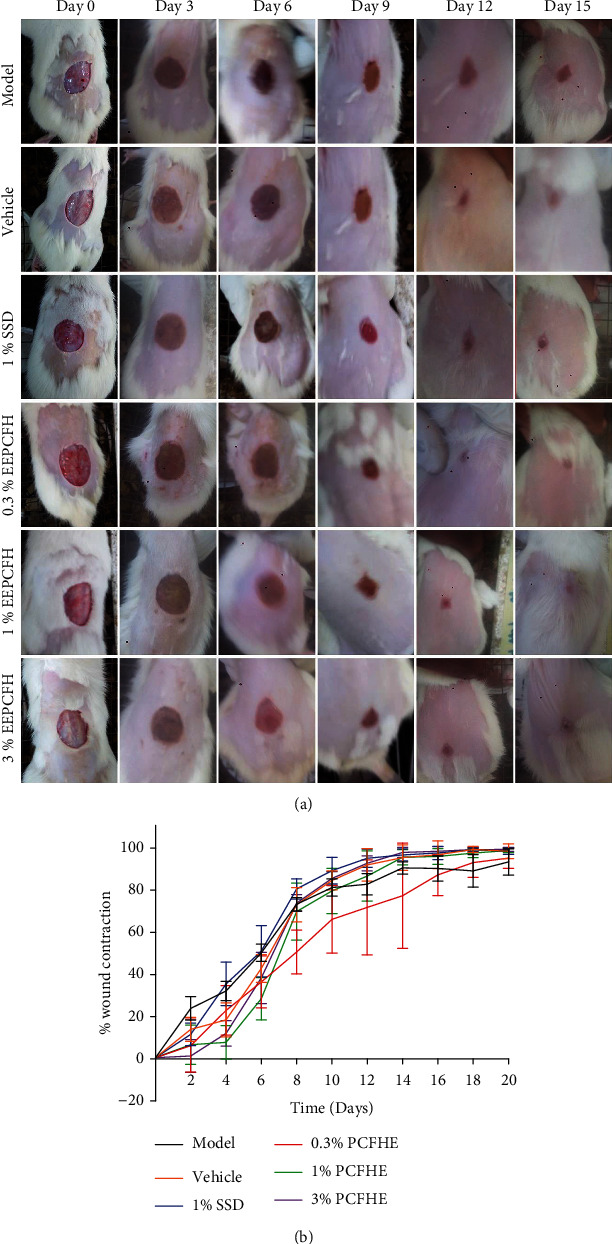
(a) Effect of PCFHE-wound healing formula on wound contraction monitored at every 72 hours during the treatment period. (b) Effect of *P. clappertoniana* fruit husk extract- (PCFHE-) reconstituted wound healing formula on rate of wound contraction in excisional-wounded rats. Data are expressed as mean%wound contraction ± SD, *n* = 5. PCFHE: *P. clappertoniana* fruit husk extract.

After establishment of excision wounds in rats, followed by daily topical treatment, it was observed that the period of epithelialization in model rats (untreated excisional-wounded rats) was higher compared to positive control group (1% SSD-treated rats). However, the period of epithelialization in excisional-wounded rats treated with PCFH wound healing formula was lower compared to both model and positive control groups, particularly the 1% and 3% PCFH groups (Figure 3). The rate of wound contraction was slower in model rats compared to positive control group (1% SSD-treated rats). However, the rate of wound contraction was improved in rats treated with PCFH-reconstituted wound healing formula compared to model and positive control groups (Figure 4).

### 3.3. Effects of PCFHE-Reconstituted Wound Healing Formula on Histology of Healed Tissue

**Figure 5:**
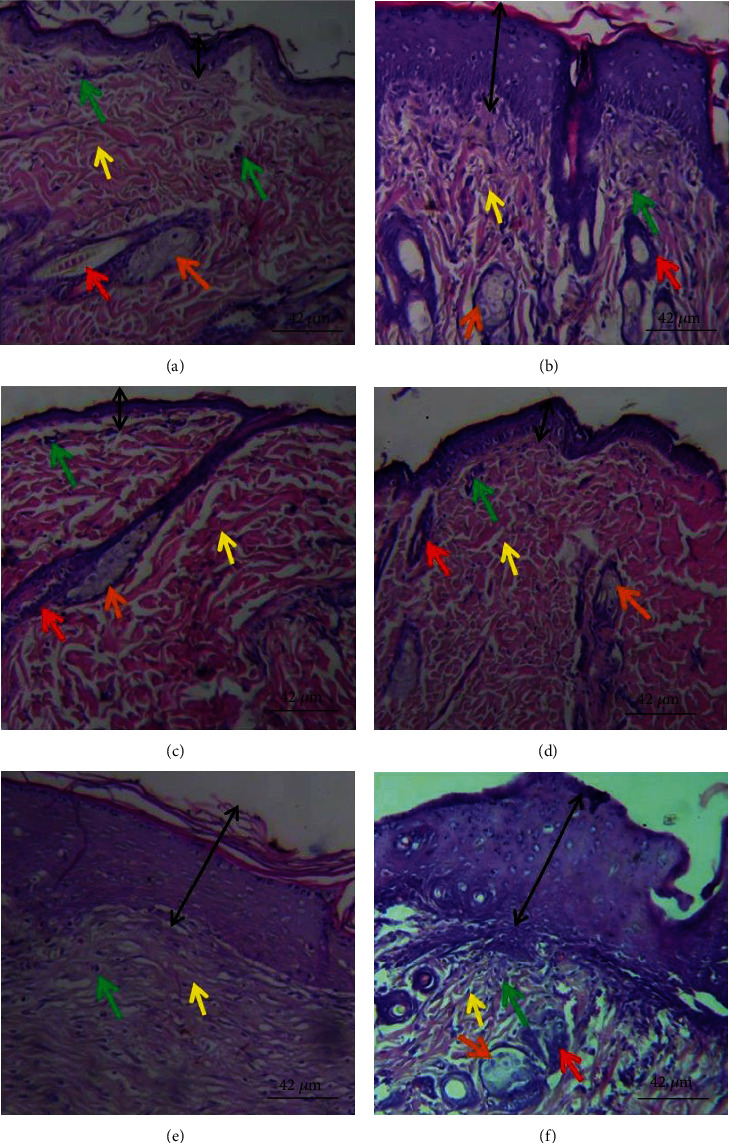
Histological section of healed wound tissue showing epidermis (black double headed arrow) and dermis. (a) Thin epidermis and slightly fibrotic granulation dermis with mild eosinophilia of collagen fibers, moderate inflammatory cell infiltration, and few sebaceous glands. (b) Thick epidermis and slightly fibrotic granulation dermis with mild eosinophilia of collagen fibers, severe inflammatory cell infiltration, coagulation in the hair follicle, and moderate abundance of sebaceous glands. (c) Thin epidermis and loose granulation dermis with widespread eosinophilic collagen fibers and moderate inflammatory cell infiltration, widespread coagulation in the hair follicle, very few hair follicle, and sebaceous glands. (d) Thin epidermis and slightly fibrotic granulation dermis with focal eosinophilia of collagen fibers, very few inflammatory cell infiltrations, and coagulation in the hair follicle. (e) Thick epidermis with appreciable keratin layer. Dermis is fibrotic with few inflammatory cell infiltration, absence of hair follicle, and sebaceous gland. (f) Thick epidermis and loose granulation dermis with moderate inflammatory cell infiltration and few sebaceous glands. Magnification: ×400. (a) Model, (b) vehicle, (c) 1% SSD, (d) 0.3% PCFHE, (e) 1% PCFHE, and (f) 3% PCFHE. PCFHE: *P. clappertoniana* fruit husk extract; SSD: silver sulfadiazine.

**Figure 6:**
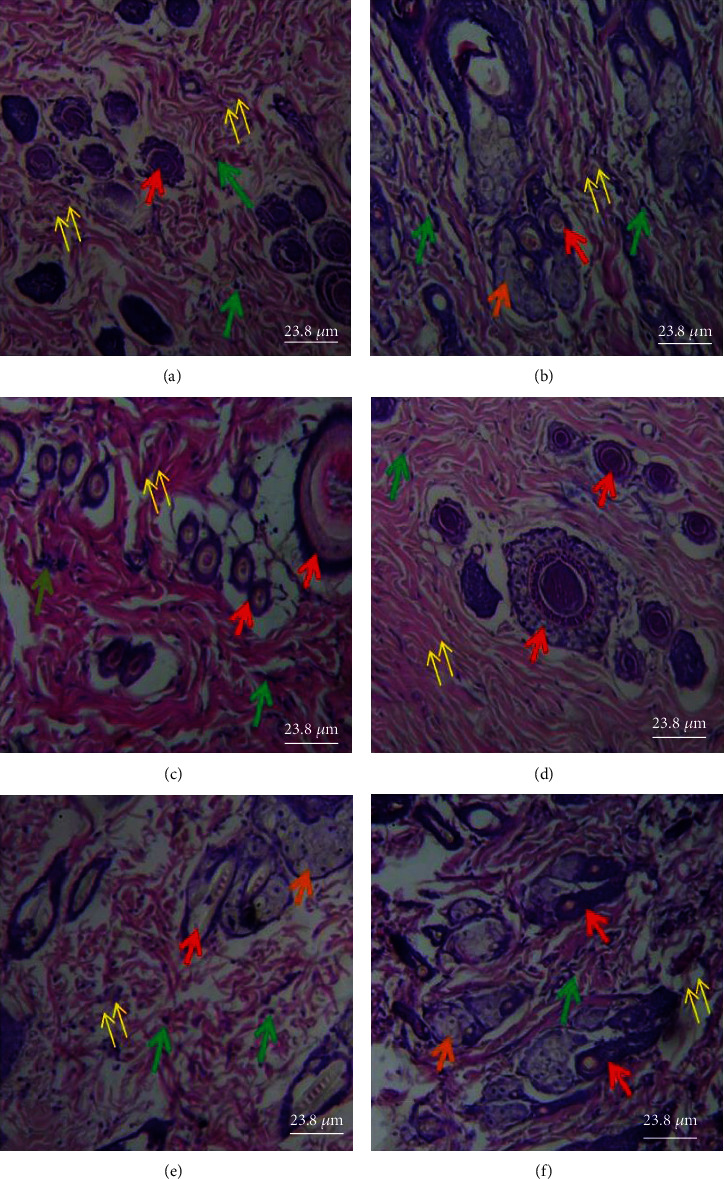
Histological section of the dermis showing pilosebaceous unit components [hair follicles (red arrow), sebaceous glands (orange arrows)], granulation tissue (yellow arrows), and inflammatory cells (green arrows). (a) Several small hair follicles but no sebaceous glands and loose granulation tissue with moderate inflammatory cells infiltration. (b) Variable-sized hair follicles, large sebaceous glands, and loose granulation tissue with few inflammatory cell infiltrations. (c) Several small hair follicles with perifollicular vasodilatation, no sebaceous glands, loose granulation tissue with eosinophilic collagen fibers, and moderate inflammatory cell infiltration. (d) Several hair follicles of variable sizes and with perifollicular vasodilatation, no sebaceous glands, and fibrotic granular tissue with no obvious inflammatory cell infiltration. (e) Less basophilic hair follicles, few sebaceous glands, and very loose granulation tissue with moderate inflammation cell infiltration. (f) Several small hair follicles and numerous sebaceous glands and loose granulation tissue with moderate inflammatory cell infiltration. Magnification: ×400. (a) Model, (b) vehicle, (c) 1% SSD, (d) 0.3% PCFHE, (e) 1% PCFHE, and (f) 3% PCFHE. PCFHE: *P. clappertoniana* fruit husk extract; SSD: silver sulfadiazine.

**Figure 7:**
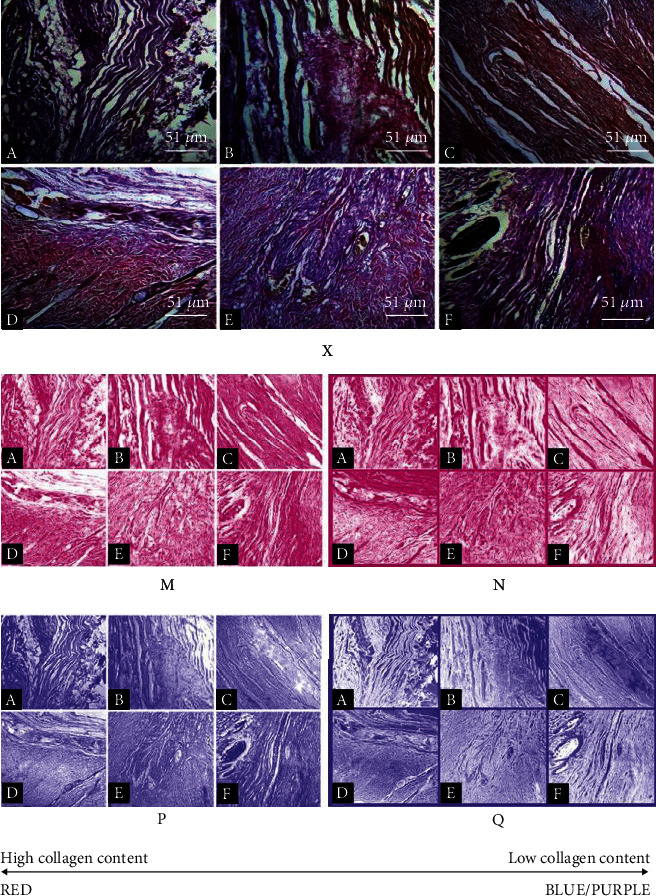
Image processing workflow of micrographs of healed wound tissues micrographs for collagen quantification. X represents original micrograph. M and P represent processed images after colour deconvolution. N and Q represent the inverted forms of M and P, respectively. Magnification: ×400. (a) Model, (b) vehicle, (c) 1% SSD, (d) 0.3% PCFHE, (e) 1% PCFHE, and (f) 3% PCFHE. PCFHE: *P. clappertoniana* fruit husk extract; SSD: silver sulfadiazine.

Figure 5 shows that model group demonstrated thin epidermis (black double-headed arrow) and slightly fibrotic granulation dermis with mild eosinophilia of collagen fibers, moderate inflammatory cell infiltration, and few sebaceous glands relative to vehicle group (thick epidermis and slightly fibrotic granulation dermis with mild eosinophilia of collagen fibers, severe inflammatory cell infiltration, coagulation in the hair follicle, moderate abundance of sebaceous glands), positive control group (shows thin epidermis and loose granulation dermis with widespread eosinophilic collagen fibers and moderate inflammatory cell infiltration (Table 4), widespread coagulation in the hair follicle, and very few hair follicle and sebaceous glands), 0.3% PCFHE (shows thin epidermis and slightly fibrotic granulation dermis with focal eosinophilia of collagen fibers, very few inflammatory cell infiltration, and coagulation in the hair follicle), 1% PCFHE (shows thick epidermis with appreciable keratin layer, fibrotic dermis with few inflammatory cell infiltration, and absence of hair follicle and sebaceous gland), and 3% PCFHE (shows thick epidermis and loose granulation dermis with moderate inflammatory cell infiltration and few sebaceous glands).  Figure 6 shows that model group demonstrated several small hair follicles (red arrow) but no sebaceous glands (orange arrow), and loose granulation tissue (yellow arrow) with moderate inflammatory cell (green arrow) infiltration, compared to vehicle group (shows variable sized hair follicles, large sebaceous glands, and loose granulation tissue with few inflammatory cell infiltrations), positive control (shows several small hair follicles with perifollicular vasodilatation, no sebaceous glands, loose granulation tissue with eosinophilic collagen fibers, and moderate inflammatory cell infiltration), 0.3% PCFHE group (shows several hair follicles of variable sizes and with perifollicular vasodilatation, no sebaceous glands, and fibrotic granular tissue with no obvious inflammatory cell infiltration), 1% PCFHE group (shows less basophilic hair follicles, few sebaceous glands, and very loose granulation tissue with moderate inflammation cell infiltration), and 3% PCFHE group (shows several small hair follicles and numerous sebaceous glands and loose granulation tissue with moderate inflammatory cell infiltration).  Figure 7 and Table 5 show that PCFHE-reconstituted wound healing formula treatment demonstrated increased collagen content compared to model and positive control groups.

### 3.4. Effects of PCFH Wound Healing Formula on Dermal Toxicity

Dermal toxicity was determined by erythema and oedema assessments after exposure of shaved skin area of rats to PCFHE and then monitored for skin reactions. Compared to negative control (NaOH-treated rats), topical exposure of shaved skin areas to PCFHE elicited no skin reactions as shown by zero erythema and oedema indices (Table 6 and S1).

### 3.5. Effect of PCFHE on the Growth of Test Organisms

PCFHE demonstrated concentration-dependent growth inhibition on *Pseudomonas aeruginosa*, *Klebsiella pneumoniae*, *Candida albicans*, and *Escherichia coli* but not *Staphylococcus aureus* comparable to standard antibiotics (ciprofloxacin and fluconazole) (Table 7). The potency of PCFHE as determined by MIC values showed that PCFH has potency comparable to ciprofloxacin. Interestingly, antifungal potency of PCFHE was higher than that of fluconazole (Tables 8 and 9).

### 3.6. Effect of PCFH on Time-Kill Kinetics of Wound Contaminants

**Figure 8:**
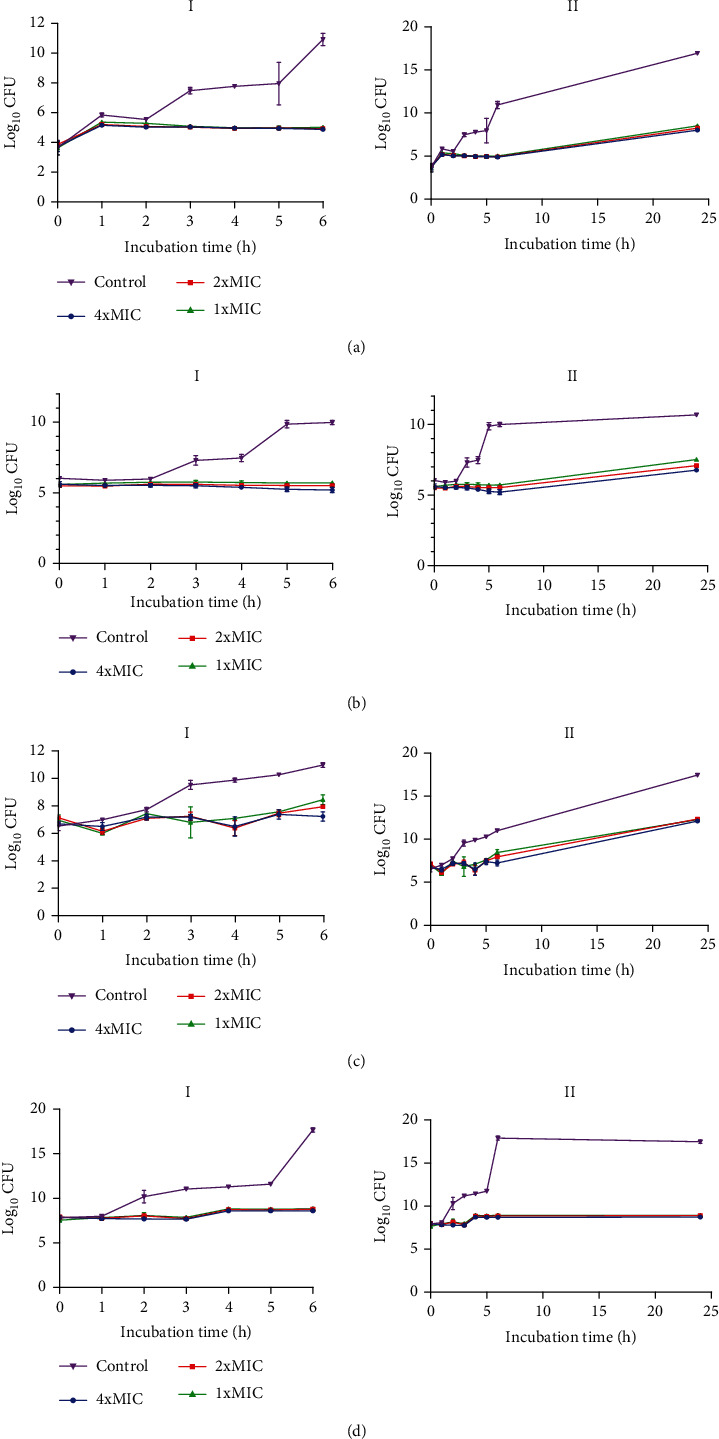
Time-kill kinetics of PCFHE against test organisms. (a) *Klebsiella pneumonia*, (b) *Pseudomonas aeruginosa*, (c) *Candida albicans*, and (d) *E. coli.* Each point is the logarithm of mean colony forming units (CFU) per mL against incubation time, *n* = 2. (I) time-kill analysis over 6 hours; (II) time-kill analysis over 24 hours. PCFHE: *P. clappertoniana* fruit husk extract.

PCFH demonstrated bacteriostatic effects on all the test organisms compared to control. At a maximum concentration of 4 × MIC, PCFH showed bacteriostatic effect lasting 24 h on the growth of *K. pneumonia*, bacteriostatic effect lasting 6 h on *P. aeruginosa*; bacteriostatic effect lasting 6 h on *E. coli*, and fungistatic effect lasting 24 h on *C. albicans* (Figure 8).

## 4. Discussion

This study assessed wound healing efficacy, antimicrobial activity, mode of antimicrobial activity, and phytochemical composition of *P. clappertoniana* fruit husk extract (PCFHE) and a PCFHE-reconstituted wound healing formula (WHF). For the first time, the present results showed that PCFHE-reconstituted wound healing formula possess wound healing efficacy in an excisional wound model in rats while PCFHE, the main component of PCFHE-WHF demonstrated Gram-selective bacteriostatic and antifungal effects against incidental and common wound contaminants. *P. clappertoniana* grows in the wild in the northern parts of most African countries including Ghana. Over the ages, the local people in northern Ghana have incorporated the use of various parts of *P. clappertoniana* in their food range and folklore. For instance, the fruit powder of *P. clappertoniana* is eaten raw or made into porridge and eaten by all age groups. Seeds of *P. clappertoniana* are processed into a local condiment called “dawadawa” which is used as a spice in the preparation of soup and other meals [15]. Interestingly, the fruit husk of *P. clappertoniana* is used as a crude wound healing agent for the treatment of sores arising from expulsion of adult guinea worms from the skin of infected people. The practice of using the fruit husk of *P. clappertoniana* to treat wounds is quite common; however, this folk claim has not been scientifically verified. Excisional wound model is one of the experimental wound models mostly used to screen medicinal plants suspected of having wound healing properties [31, 32].

Wound healing is a complex physiological process which involves tissue repair and remodeling essential for restoration of functional and structural integrity of epithelial tissues to guarantee barrier function [3, 9, 33]. During wound healing process, reepithelialization and tissue granulation are indispensable [34]. Similarly, the period of restoring the barrier function is as important as the reepithelialization process [35]. Therefore, wound contraction, epithelialization, and granulation are used as key benchmarks to assess wound healing efficacy [9]. In this study, it was observed that excision wounds treated with PCFHE-reconstituted wound healing formula improved wound contraction and the period of reepithelialization (Figures 1–3) compared to model (negative control) and positive control (silver sulfadiazine- [SSD-] treated excisional wounds). Also, from the histological assessment of healed wound tissues, it was observed that not only did PCFHE-reconstituted wound healing formula-treated wounds demonstrated thick epithelial tissues and collagen deposition in the dermis but also improved granulation and reduced inflammatory infiltration compared to model group (Figures 4–6), and this observation is not different from those observed in other plants which have demonstrated wound healing properties such as *Pistacia atlantica*, *Zataria multiflora*, *Trifolium repens*, and *Salvia officinalis* [9–12]. Inflammation and algesia are key components of the milieu that follow pathological break in the continuity of epithelial tissues. As can be deduced from the histo-micrographs (Figures 4–6), PCFHE significantly reduced inflammatory cell infiltration compared to model, suggestive of possible anti-inflammatory properties of PCFHE.

Wound contamination is one of the factors that contributes to chronic nonhealing wounds. Bacteria and to some extent fungi species colonize epithelial tissues such as the skin as normal flora. As a result, a break in the continuity of the skin characteristic of cutaneous wounds provides an opportunity for wound contamination by resistant microbial agents including bacteria and fungi species. Some microbes are naturally nonpathogenic but becomes pathogenic when they are displaced from their niche into anatomic sites including wounds. It is estimated that 10^5^ bacteria colonize the skin and could be clinically relevant in wound infection [9] and chronic nonhealing wounds [36, 37]. It is suggested that incidental cultures should be distinguished from true pathogens infecting a wound [3]. Isolation of the causative organism in an infected wound is critical for wound treatment and management. Microorganisms such as *Staphylococcus aureus* and *Pseudomonas aeruginosa* [9], as well as *E. coli*, *Candida albicans*, *Klebsiella pneumonia*, and other Gram positive and Gram negative bacteria are implicated in wound contamination and related chronic nonhealing wounds. From the present study, *Staphylococcus aureus*, *Pseudomonas aeruginosa*, *E. coli*, *Candida albicans*, and *Klebsiella pneumonia* were isolated and cultured from excisional wound swabs, and this observation in part adds credence to earlier reports implicating these microorganisms in wound infections [38]. Interestingly, all the isolated microorganisms except *Staphylococcus aureus* demonstrated significant sensitivity to increasing concentrations of PCFHE, indicating that PCFHE has Gram-selective antibacterial effect, specifically Gram-negative bias as well as antifungal effects. Mechanistically, it was observed that PCFHE exerts bacteriostatic effects against the test microorganisms over a period of 6-24 hours (Figure 7). The bioactivity of medicinal plants has always been attributed to their phytochemical composition and functional group enrichment [39].

Diverse secondary plant metabolites including alkaloids, phenolic compounds, tannins, saponins, and terpenoids just to mention but a few, demonstrate various bioactivities including antimicrobial, fibrotic, cell-cell motility, extracellular matrix (ECM) formation, and proliferative effects [18]. *P. clappertoniana* fruit husk extract (PCFHE) has demonstrated wound healing efficacy in excisional wounds in rats as well as Gram-selective bacteriostatic and antifungal activity against common wound contaminants, and it was also dermatologically safe (S1) in rats. From the present study, phenols, flavonoids, tannins, alkaloids, terpenoids, glycosides, and saponins were identified in PCFHE (Table 3). Interestingly, wound healing and antimicrobial potentials of these phyto-compounds have been demonstrated in previous studies involving medicinal plant extracts [40–42]. Mechanistically, these phyto-compounds exact biological processes including but not limited to metal chelation, free radical scavenging, and immunological regulation either individually or synergistically to promote wound healing [43]. In this study, HPLC chromatogram for PCFHE revealed 15 peaks out of which 10 were very prominent (Figure 2). The retention times ranged from 4.824 to 19.266 min (Figure 2(c)). Reference compounds, catechin and quercetin (Figures 2(a) and 2(b)), eluted at 13.138 min and 17.958 min, respectively, and this corresponded with diminished peaks in PCFHE chromatogram (13.639 min and 18.255 min) (Figures 2(a) and 2(b)). Further, colorimetric phytochemical test was used to confirm the presence of catechin and quercetin in PCFHE, indicating that the wound healing and antimicrobial effects of PCFHE are attributable to its phyto-components particularly catechin and quercetin. This assertion is in view of the confirmed wound healing, antioxidant, anti-inflammatory, and antimicrobial effects of catechin and quercetin [44, 45]. For instance, quercetin and catechins demonstrate free radical scavenging, anti-inflammatory, antioxidant, antihistamine effect, inhibition of matrix metalloproteinase, and promote collagen synthesis [46–48], and all these properties are crucially involved in wound healing process [49]. This study could have benefited from an *in vitro* wound healing model such as use of keratinocytes or other commercially available skin cells to further explore the mechanisms by which PCFHE improved the wound healing process. Also, future studies should consider isolation of catechin and quercetin as well as other phenolic compounds from PCFHE in view of their potential as natural templates for development of new wound healing agents. Notwithstanding the above limitations, the present *in vivo* demonstration of wound healing and antimicrobial effects of PCFHE provide a strong rationale for further explorative studies on *P. clappertoniana* in search for novel wound healing agents.

## 5. Conclusion

*P. clappertoniana* fruit husk has demonstrated wound healing efficacy and Gram-selective bacteriostatic and fungistatic effects against bacterial and fungal wound contaminants in excisional wounds in rats, and these observations are attributable to the phytochemical composition of PCFHE. Quercetin and catechins detected in PCFHE suggest that the mechanism by which PCFHE improved wound healing may be related to antioxidant mechanisms. Put together, these findings confirm use of *P. clappertoniana* fruit husk as a wound healing agent in Northern Ghana. Finally, the present findings present a rationale for further studies on PCFHE for development of new and easily accessible wound healing agents.

## Figures and Tables

**Table 1:** Components and formulation of PCFH-RWHF.

Components	PCFHE-RWHF (%)			Vehicle
	0.30	1	3	
	Amount (g)			
PCFHE	0.108	0.36	1.08	—
Emulsifying ointment	10.8	10.7	10.5	10.8
Purifying water	25.1	25	24.4	25.2

PCFHE: *P. clappertoniana* fruit husk extract; PCFHE-WHF: PCFHE-reconstituted wound healing formula.

**Table 2:** Erythema and oedema scoring guide for determination of primary skin irritation index [25].

Erythema	Value
No erythema	0
Very slight erythema (barely perceptible)	1
Well-defined erythema	2
Moderate to severe erythema	3
Severe erythema (beef redness)	4
Oedema formation	Value
No oedema	0
Very slight oedema (barely perceptible)	1
Slight oedema (edges of area well-defined by definite raising)	2
Moderate oedema (raised approximately 1 mm)	3
Severe oedema (extending beyond the area of exposure)	4

**Table 3:** Preliminary qualitative phytochemical assessment of PCFHE.

Phyto-constituents	Test	Results
Alkaloids	Dragendroff	+
	Meyer	+
Phenols	Fecl3	+
Flavonoids	Lead acetate	+
Tannins	Gelatin	+
Glycosides	Keller-Kiliani test	+
Saponin	Frothing test	-
Terpenoids	Salkowski test	+
Quinones	Bontrager's test	-

+: present; -: not present; PCFHE: *P. clappertoniana* fruit husk extract.

**Table 4:** Effect of *P. clappertoniana* fruit husk extract- (PCFH-) reconstituted wound healing formula (RWHF) on histological integrity of healed wound tissues after complete epithelialization.

Histopathological observation	Treatment					
	Model	Vehicle	1% SSD	0.3% PCFHE	1% PCFHE	3% PCFHE
Epithelium	++	++	++	++	++	+
Fibroblast	+	+	++	++	++	++
Collagen	++	+	++	+	++	++
Inflammation	++	++	+	+	+	+
Vascularization	++	++	++	+	++	++

Grading scale: collagen (+: not enough collagen; ++: enough collagen; +++: keloid formation); epithelia (+: thin; ++: thick; +++: thick enough); inflammation (+: few scattered lymphocytes; ++: enough lymphocytes); vascularization (+: few blood vessels; ++: many blood vessels); fibroblast (+: less dense; ++: denser). PCFHE: *P. clappertoniana* fruit husk extract; SSD: silver sulfadiazine.

**Table 5:** Quantification of collagen content in picrosirius red- (red) and Van Gieson- (blue) stained healed wound tissues.

Treatment groups	Red (%)	Blue (%)	Average	Red/blue ratio
Model	48.41	61.35	54.88	0.7891
Vehicle	57.36	51.55	54.46	1.1127
1% SSD	70.93	51.93	61.43	1.3659
PCFH (%)				
0.3	48.58	48.72	48.65	0.9971
1.0	52.89	65.73	59.31	0.8047
3.0	61.66	67.89	64.78	0.9082

PCFHE: *P. clappertoniana* fruit husk extract; SSD: silver sulfadiazine.

**Table 6:** Dermal toxicity assessment of *P. clappertoniana* fruit husk extract- (PCFHE-) reconstituted wound healing formula (RWHF) topically applied to shaved skin areas of rats.

Animal	Period after treatment					Erythema index	Oedema index
	0 h	6 h	24 h	48 h	72 h		
1	No behavioral change, i.e., in locomotion, no defecation,	No behavioral change during their normal activities—feeding and drinking water	No sign of oedema, erythema	No sign of oedema, erythema	No sign of oedema, erythema	0	0
2	No behavioral change, i.e., in locomotion, no defecation	No behavioral change during their normal activities—feeding and drinking water	No sign of oedema, erythema	No sign of oedema, erythema	No sign of oedema, erythema	0	0
3	No behavioral change, i.e., in locomotion, no defecation	No behavioral change during their normal activities—feeding and drinking water	No sign of oedema, erythema	No sign of oedema, erythema	No sign of oedema, erythema	0	0
Positive control 1 g (NaOH)	No behavioural change, i.e., in locomotion, no defecation	No behavioural change during their normal activities—feeding and drinking water	Oedema, erythema seen	Oedema, erythema seen	Oedema, erythema seen	4	2

No erythema/no oedema = 0; very light erythema (barely perceptible)/very light oedema (barely perceptible) = 1; well-defined erythema/slight oedema (edges of area well-defined by definite raising) = 2; moderate to severe erythema/moderate oedema (raised approximately 1 mm) = 3; severe erythema (beef redness)/(extending beyond the area of exposure) = 4; *P. clappertoniana* fruit husk extract (PCFHE).

**Table 7:** Effect of *P. clappertoniana* fruit husk extract (PCFHE) on growth of test microorganisms.

Drugs (mg/mL)	Organisms (zone of inhibition [mm])				
	*Pseudomonas aeruginosa*	*Klebsiella pneumoniae*	*Candida albicans*	*Escherichia coli*	*Staphylococcus aureus*
PCFH					
12.5	2.67 ± 4.6	2.67 ± 4.6	3.33 ± 5.8	7.67 ± 0.6	-
25	9.00 ± 0.0	7.00 ± 6.2	11.00 ± 2.0	9.33 ± 0.6	-
50	10.00 ± 0.0	11.33 ± 0.6	12.00 ± 2.6	10.67 ± 0.6	-
100	11.67 ± 1.2	12.67 ± 0.6	13.33 ± 1.5	12.00 ± 1.0	-
200	13.67 ± 2.1	13.0 ± 1.7	14.33 ± 2.5	14.33 ± 0.6	-
Ciprofloxacin^∗^	24.00 ± 3.0	24.67 ± 1.2	24.67 ± 1.2	27.33 ± 1.2	23.67 ± 2.5
Fluconazole^#^	-	-	27.33 ± 3.1	-	-

Values are mean ± SD, *n* = 3, diameters of zones of inhibition after incubation of test organisms with increasing concentrations of PCFHE. PCFHE: *P. clappertoniana* fruit husk extract; -: no zone of inhibition or not applicable; ^∗^(0.01 mg/mL); ^#^(1 mg/mL).

**Table 8:** Minimum inhibitory concentration (MIC) of *P. clappertoniana* fruit husk extract (PCFHE) for the various test microorganisms.

Test microorganisms	Concentrations (mg/mL)							
	40	20	10	5	2.5	1.25	0.625	0.3125
PCFHE								
*Pseudomonas aeruginosa*	-	-	-	-	-	+	+	+
*Klebsiella pneumoniae*	-	-	-	-	-	-	+	+
*Escherichia coli*	-	-	-	-	-	+	+	+
*Candida albicans*	-	-	-	-	-	+	+	+
Ciprofloxacin								
*Pseudomonas aeruginosa*	-	-	-	-	-	-	+	+
*Klebsiella pneumoniae*	-	-	-	-	-	+	+	+
*Escherichia coli*	-	-	-	-	-	-	+	+
Fluconazole								
*Candida albicans*	-	-	-	+	+	+	+	+

+: growth; -: no growth; PCFHE: *P. clappertoniana* fruit husk extract.

**Table 9:** Minimum inhibitory concentration (MIC), minimum bactericidal concentration (MBC), and minimum fungicidal concentration (MFC) estimates for *P. clappertoniana* fruit husk extract (PCFHE) against wound contaminants.

Test microorganisms	Concentrations (mg/mL)		
	PCFHE	Ciprofloxacin	Fluconazole
MIC			
*Pseudomonas aeruginosa*	2.50	2.50	-
*Klebsiella pneumoniae*	1.25	2.50	-
*Escherichia coli*	2.50	1.25	-
*Candida albicans*	2.50	-	10
MBC			
*Pseudomonas aeruginosa*	40	1.25	
*Klebsiella pneumoniae*	20	10.0	
*Escherichia coli*	40	5.0	
MFC			
*Candida albicans*	40	-	20

-: not applicable. PCFH: *P. clappertoniana* fruit husk extract; MBC: minimum bactericidal concentration; MFC: minimum fungicidal concentration; MIC: minimum inhibitory concentration.

## Data Availability

The data is available at http://ir.ucc.edu.gh/xmlui.

## Supplementary Materials

Supplementary Materials S1. Degree of skin allergic reactions after topical exposure of shaved dorsal skin area of rats to PCFHE-WHF and NaOH. After topical application of PCFHE-WHF and NaOH, skin areas were monitored for allergic skin reactions over a period of 48 hours. PCFHE-WHF: Parkia clappertoniana fruit husk extract reconstituted wound healing formula; NaOH: sodium hydroxide.

## References

[1] Costantini E., Sinjari B., Falasca K. (2021). Assessment of the vanillin anti-inflammatory and regenerative potentials in inflamed primary human gingival fibroblast. *Mediators of Inflammation*.

[2] Olsson M., Järbrink K., Divakar U. (2019). The humanistic and economic burden of chronic wounds: a systematic review. *Wound Repair and Regeneration*.

[3] Han G., Ceilley R. (2017). Chronic wound healing: a review of current management and treatments. *Advances in Therapy*.

[4] Paquette D., Falanga V. (2002). Leg ulcers. *Clinics in Geriatric Medicine*.

[5] DeLeo F. R., Chambers H. F. (2009). Reemergence of antibiotic-resistant Staphylococcus aureus in the genomics era. *The Journal of Clinical Investigation*.

[6] Komarcević A. (2000). The modern approach to wound treatment. *Medicinski Pregled*.

[7] Game F. L., Apelqvist J., Attinger C. (2016). Effectiveness of interventions to enhance healing of chronic ulcers of the foot in diabetes: a systematic review. *Diabetes/Metabolism Research and Reviews*.

[8] Hofmann A. T., Neumann S., Ferguson J., Redl H., Mittermayr R. (2017). A rodent excision model for ischemia-impaired wound healing. *Tissue Engineering. Part C, Methods*.

[9] Farahpour M. R., Pirkhezr E., Ashrafian A., Sonboli A. (2020). Accelerated healing by topical administration of _Salvia officinalis_ essential oil on _Pseudomonas aeruginosa_ and _Staphylococcus aureus_ infected wound model. *Biomedicine & Pharmacotherapy*.

[10] Habibi Zadeh S. K., Farahpour M. R., Kar H. H. (2020). The effect of topical administration of an ointment prepared from Trifolium repens hydroethanolic extract on the acceleration of excisional cutaneous wound healing. *Wounds*.

[11] Farahpour M. R., Mirzakhani N., Doostmohammadi J., Ebrahimzadeh M. (2015). Hydroethanolic _Pistacia atlantica_ hulls extract improved wound healing process; evidence for mast cells infiltration, angiogenesis and RNA stability. *International Journal of Surgery*.

[12] Farahpour M. R., Sheikh S., Kafshdooz E., Sonboli A. (2021). Accelerative effect of topical Zataria multiflora essential oil against infected wound model by modulating inflammation, angiogenesis, and collagen biosynthesis. *Pharmaceutical Biology*.

[13] Dardmah F., Farahpour M. R. (2021). Quercus infectoria gall extract aids wound healing in a streptozocin-induced diabetic mouse model. *Journal of Wound Care*.

[14] Dinda B., Mohanta B. C., Debnath S. (2009). Iridoid glucosides from leaves and stem barks of *Parkia javanica*. *Journal of Asian Natural Products Research*.

[15] Boye A., Boampong V. A., Takyi N., Martey O. (2016). Assessment of an aqueous seed extract of _Parkia clappertoniana_ on reproductive performance and toxicity in rodents. *Journal of Ethnopharmacology*.

[16] Lemmich E., Adewunmi C. O., Furu P., Kristensen A., Larsen L., Olsen C. E. (1996). 5-deoxyflavones from _Parkia clappertoniana_. *Phytochemistry*.

[17] Boye A. (2014). Nephroprotective and curative assessment of an aqueous seed extract of Parkia clappertoniana keay in gentamicin-induced renal damage in Sprague-dawley rats. *European Journal of Medicinal Plants*.

[18] Saleh M. S., Jalil J., Zainalabidin S., Asmadi A. Y., Mustafa N. H., Kamisah Y. (2021). Genus Parkia: phytochemical, medicinal uses, and pharmacological properties. *International Journal of Molecular Sciences*.

[19] Banwo G., Abdullahi I., Duguryil M. (2004). The antimicrobial activity of the stem-bark and leaf of Parkia clappertoniana Keay family Leguminosae against selected microorganisms. *Nigerian Journal of Pharmaceutical Research*.

[20] Patrick-Iwuanyanwu K., Wegwu M., Okiyi J. (2010). Hepatoprotective effects of African locust bean (Parkia clappertoniana) and negro pepper (Xylopia aethiopica) in CCl4-induced liver damage in Wistar albino rats. *International Journal of Pharmacology*.

[21] Li W., Zhang X., Chen R. (2020). HPLC fingerprint analysis of _Phyllanthus emblica_ ethanol extract and their antioxidant and anti-inflammatory properties. *Journal of Ethnopharmacology*.

[22] Rhea L., Dunnwald M. (2020). Murine excisional wound healing model and histological morphometric wound analysis. *Journal of Visualized Experiments*.

[23] George M., Morgan J. B., Glock R. D. (1995). Injection-site lesions: incidence, tissue histology, collagen concentration, and muscle tenderness in beef rounds. *Journal of Animal Science*.

[24] Nair S., Mathew M., Sreena K. (2012). Evaluation of skin irritation of herbal antioxidant cream. *Asian J Biochem Pharm Res*.

[25] Nair S. S., Mathew M., Sreena K. (2012). Asian Journal of Biochemical and Pharmaceutical Research. *Asian Journal of Biochemical and Pharmaceutical Research*.

[26] Boakye Y., Agyare C., Hensel A. (2016). Anti-infective properties and time-kill kinetics of Phyllanthus muellerianus and its major constituent, geraniin. *Medicinal Chemistry*.

[27] Nigussie D., Makonnen E., Legesse B. A., Fekadu A., Davey G. (2020). Antimicrobial susceptibility of bacteria isolated from the infected wounds of patients with lymphoedema in East Wollega, Ethiopia. *Transactions of the Royal Society of Tropical Medicine and Hygiene*.

[28] Wiegand I., Hilpert K., Hancock R. E. (2008). Agar and broth dilution methods to determine the minimal inhibitory concentration (MIC) of antimicrobial substances. *Nature Protocols*.

[29] Hackel M. A., Karlowsky J. A., Dressel D., Sahm D. F. (2017). Determination of disk diffusion and MIC quality control ranges for nafithromycin (WCK 4873), a new lactone-ketolide. *Journal of Clinical Microbiology*.

[30] Brennan-Krohn T., Kirby J. E. (2019). Antimicrobial synergy testing by the inkjet printer-assisted automated checkerboard array and the manual time-kill method. *Journal of Visualized Experiments*.

[31] Masson-Meyers D. S., Andrade T. A. M., Caetano G. F. (2020). Experimental models and methods for cutaneous wound healing assessment. *International Journal of Experimental Pathology*.

[32] Thakur R., Jain N., Pathak R., Sandhu S. S. (2011). Practices in wound healing studies of plants. *Evidence-based Complementary and Alternative Medicine*.

[33] Costantini E., Sinjari B., D’Angelo C., Murmura G., Reale M., Caputi S. (2019). Human gingival fibroblasts exposed to extremely low-frequency electromagnetic fields: in vitro model of wound-healing improvement. *International Journal of Molecular Sciences*.

[34] Galiano R. D., Michaels, V J., Dobryansky M., Levine J. P., Gurtner G. C. (2004). Quantitative and reproducible murine model of excisional wound healing. *Wound Repair and Regeneration*.

[35] Daemi A., Lotfi M., Farahpour M. R., Oryan A., Ghayour S. J., Sonboli A. (2019). Topical application of Cinnamomum hydroethanolic extract improves wound healing by enhancing re-epithelialization and keratin biosynthesis in streptozotocin-induced diabetic mice. *Pharmaceutical Biology*.

[36] Calà C., Amodio E., di Carlo E., Virruso R., Fasciana T., Giammanco A. (2015). Biofilm production in Staphylococcus epidermidis strains, isolated from the skin of hospitalized patients: genetic and phenotypic characteristics. *The New Microbiologica*.

[37] Tarusha L., Paoletti S., Travan A., Marsich E. (2018). Alginate membranes loaded with hyaluronic acid and silver nanoparticles to foster tissue healing and to control bacterial contamination of non-healing wounds. *Journal of Materials Science. Materials in Medicine*.

[38] Atiyeh B. S., Dibo S. A., Hayek S. N. (2009). Wound cleansing, topical antiseptics and wound healing. *International Wound Journal*.

[39] Acheampong D. O., Baffour I. K., Atsu Barku V. Y., Addo J. K., Essuman M. A., Boye A. (2021). Zanthoxylum zanthoxyloides alkaloidal extract improves CCl4-induced hepatocellular carcinoma-like phenotypes in rats. *Evidence-based Complementary and Alternative Medicine*.

[40] Rane M. M., Mengi S. A. (2003). Comparative effect of oral administration and topical application of alcoholic extract of _Terminalia arjuna_ bark on incision and excision wounds in rats. *Fitoterapia*.

[41] Murugesu S., Selamat J., Perumal V. (2021). Phytochemistry, pharmacological properties, and recent applications of Ficus benghalensis and Ficus religiosa. *Plants (Basel)*.

[42] Ganie S. A., Yadav S. S. (2014). Holoptelea integrifolia (Roxb.) Planch: a review of its ethnobotany, pharmacology, and phytochemistry. *BioMed Research International*.

[43] Das U., Behera S. S., Pramanik K. (2017). Ethno-herbal-medico in wound repair: an incisive review. *Phytotherapy Research*.

[44] Chaniad P., Tewtrakul S., Sudsai T., Langyanai S., Kaewdana K. (2020). Anti-inflammatory, wound healing and antioxidant potential of compounds from Dioscorea bulbifera L. bulbils. *PLoS One*.

[45] Moulaoui K., Caddeo C., Manca M. L. (2015). Identification and nanoentrapment of polyphenolic phytocomplex from _Fraxinus angustifolia_ : _in vitro_ and _in vivo_ wound healing potential. *European Journal of Medicinal Chemistry*.

[46] Polerà N., Badolato M., Perri F., Carullo G., Aiello F. (2019). Quercetin and its natural sources in wound healing management. *Current Medicinal Chemistry*.

[47] Kant V., Jangir B. L., Kumar V., Nigam A., Sharma V. (2020). Quercetin accelerated cutaneous wound healing in rats by modulation of different cytokines and growth factors. *Growth Factors*.

[48] Chittasupho C., Manthaisong A., Okonogi S., Tadtong S., Samee W. (2022). Effects of quercetin and curcumin combination on antibacterial, antioxidant, in vitro wound healing and migration of human dermal fibroblast cells. *International Journal of Molecular Sciences*.

[49] Rodrigues M., Kosaric N., Bonham C. A., Gurtner G. C. (2019). Wound healing: a cellular perspective. *Physiological Reviews*.

