# Supplementary material for: Wound Healing Properties and Antimicrobial Effects of Parkia clappertoniana Keay Fruit Husk Extract in a Rat Excisional Wound Model
Source: Biomed Res Int. 2022 Jul 23;2022:9709365. doi: 10.1155/2022/9709365 (PMC9338854; doi:10.1155/2022/9709365)
Supplement: Supplementary Materials — S1. Degree of skin allergic reactions after topical exposure of shaved dorsal skin area of rats to PCFHE-WHF and NaOH. After topical application of PCFHE-WHF and NaOH, skin areas were monitored for allergic skin reactions over a period of 48 hours. PCFHE-WHF: Parkia clappertoniana fruit husk extract reconstituted wound healing formula; NaOH: sodium hydroxide. [file 9709365.f1.pdf]

Positive control (NaOH)

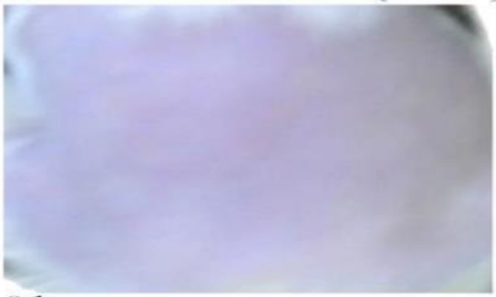

0 hour

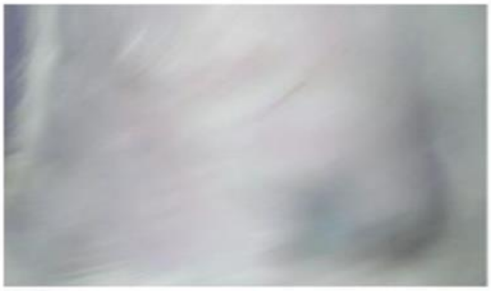

6 hours

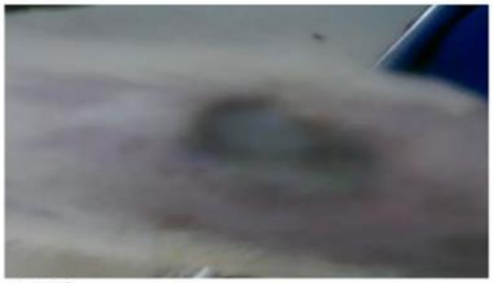

24 hours

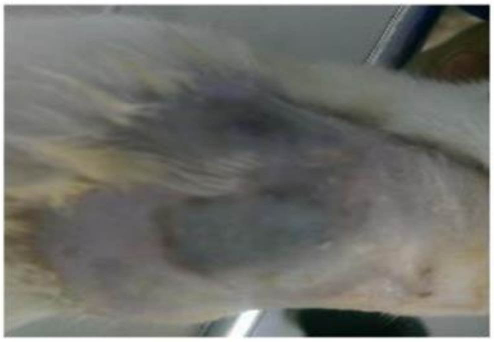

48 hours

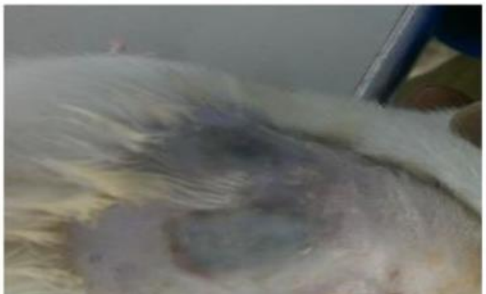

PCFHE

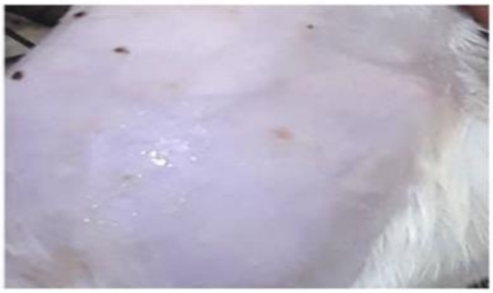

0 hour

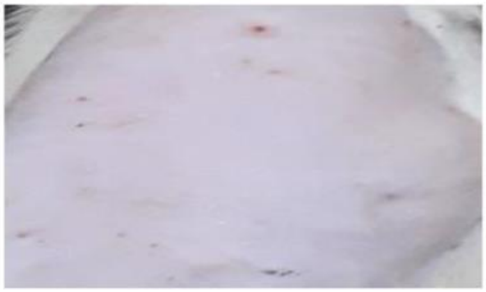

6 hours

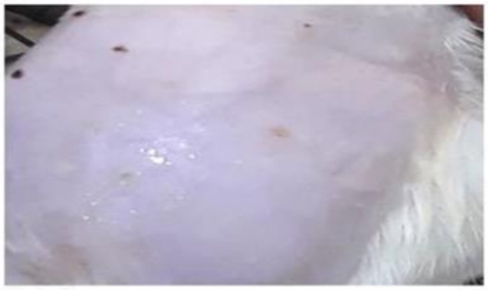

24 hours

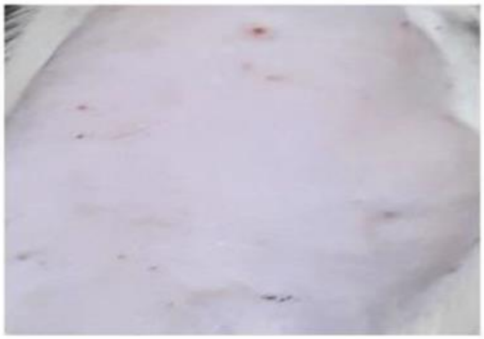

48 hours

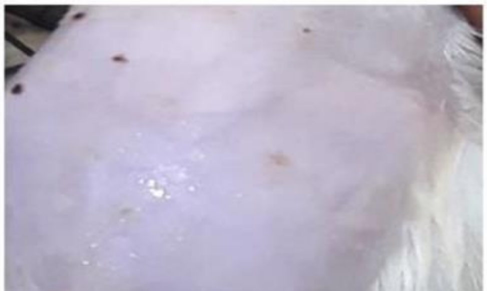

S1. Degree of skin allergic reactions after topical exposure of shaved dorsal skin area of rats to PCFHE-WHF and NaOH. After topical application skin areas were monitored every 24 hours for 3 days. PCFHE-WHF – *Parkia clappertoniana* fruit husk extract reconstituted wound healing formula; NaOH – Sodium hydroxide.
